# SPAD dynamics in maize crop with precision nitrogen management under rain-fed and irrigated conditions

**DOI:** 10.1038/s41598-025-05255-y

**Published:** 2025-07-02

**Authors:** K. Shivashankar, M. P. Potdar, Sandeep Gawdiya, Aishwarya Golshetti, Aditya Kamalakar Kanade, Gurupada Balol, D. P. Biradar, K. K. Math, Nadhir Al-Ansari, Salah El-Hendawy, Mohamed A. Mattar, Ali Salem

**Affiliations:** 1 Department of Agronomy, College of Agriculture, UAS, Dharwad, 580005 India; 2 School of Agriculture, Galgotias University, Greater Noida, Uttar Pradesh, 203201 India; 3 Department of Agronomy, College of Agriculture, V C Farm Mandya, University of Agricultural Sciences, Bengaluru, India; 4 Department of Agronomy, Mahatma Phule Krishi Vidyapith, Rahuri, 413722 Maharashtra India; 5 AICRP on MULLaRP, UAS, Dharwad, 580005 India; 6 Department of Soil Science and Agriculture Chemistry, College of Agriculture, UAS, Dharwad, India; 7 Department of Civil, Environmental, and Natural Resources Engineering, Lulea University of Technology, Lulea, 97187 Sweden; 8 Department of Plant Production, College of Food and Agricultural Sciences, King Saud University, P.O. Box 2460, Riyadh 11451, Saudi Arabia; 9 Department of Agricultural Engineering, College of Food and Agriculture Sciences, King Saud University, P.O. Box 2460, Riyadh 11451, Saudi Arabia; 10 Civil Engineering Department, Faculty of Engineering, Minia University, Minia, 61111 Egypt; 11 Structural Diagnostics and Analysis Research Group, Faculty of Engineering and Information Technology, University of Pécs, Pécs 7622, Hungary

**Keywords:** Precision nitrogen management, SPAD dynamics, Grain yield, Correlation analysis, Multivariate analysis, Agroecology, Agroecology

## Abstract

Real-time monitoring of canopy chlorophyll content is crucial for understanding crop growth and guiding precision agricultural management. The SPAD chlorophyll meter is a valuable tool for assessing nitrogen status in maize (*Zea mays* L.), a key cereal crop used for food, feed, and biofuels. Efficient nitrogen management is essential to maximize maize yield, particularly under varying water regimes. A study conducted over two years (2020–2021) utilized a strip plot design to investigate the spatiotemporal dynamics of SPAD readings and their correlation with maize yield under rainfed (M1) and irrigated (M2) conditions. Eight precision nitrogen management practices were implemented, including SPAD at sufficiency index and Green Seeker at response index, achieving ranges of 86–100% and 1.11–1.41, respectively. The findings revealed that irrigated maize produced significantly higher grain yields (6347 kg ha^–1^) compared to rainfed maize (5262 kg ha^–1^). The highest yield (9508.2 kg ha^–1^) was achieved when nitrogen was applied at a sufficiency index of 96–100%. The correlation between SPAD values and grain yield was strongest at reproductive stages (VT and R4), with R² values of 0.99 and 0.98 under rainfed conditions. In irrigated conditions, R² values ranged from 0.95 to 0.96 for earlier growth stages (V10, V12, VT, and R4). Multivariate analysis indicated critical management stages for optimizing yields in both conditions. Overall, SPAD-based nitrogen management strategies have the potential to enhance maize yields and resource efficiency while informing the development of sophisticated monitoring tools for real-time crop management.

## Introduction

Cereals provide a crucial food security globally^1^. Maize (*Zea mays* L.) is particularly important due to its C_4_ photosynthetic pathway, allowing it to thrive under various temperatures, earning the nickname ‘super plant’^2^. Maize is a versatile, serving as food, animal feed, and a major feedstock for biofuel production, especially bioethanol^3^. Hence it is called a “renewable resource”^4^. Maize is consumed as cobs or kernels and is processed into products like breakfast cereals, snacks, tortillas, sweeteners, beer, and other alcoholic beverages. Its significance is growing in renewable biofuels production, besides traditional uses. The outlook for corn appears promising as it gains significance in the production of value-added industrial goods, such as renewable biofuels, in addition to its traditional uses in feed and food production.

Of all the cereals, maize in general and hybrids in particular are most receptive to supplemental nutrients. However, the amount of applied nutrients should match with the plant’s demand and soil supply capacity to achieve desirable yields^5^. Controlling nitrogen (N) is the most crucial in maize productivity^6^. All amino acids require nitrogen as an important component. Nitrogen plays a vital role as a component of chlorophyll, the apparatus of photosynthesis, besides imparts the dark green colour to leaves^7^. The leaves of plants that receive enough nitrogen will be a deep green colour and will grow rapidly^8^. The dynamics of nitrogen supply to plants furnish the necessary conditions for the production of chlorophyll, influencing the vibrant green color in plants; therefore, the spectral characteristics of plant leaves can be used as an index for developing a real-time and non-destructive estimation of maize N status^9^.

One of the most promising methods involves leveraging proximal and remote sensing technologies, such as ground-based active optical sensors, that accurately remarks the nitrogen status of crops^10^. Among the currently developed tools, SPAD chlorophyll meter offers an opportunities in making significant decisions on nutrient management by using spectral signatures of the plant^11^. However, to synchronize nitrogen application with crop demand need to conceptualise the relationship between nitrogen and the spectral properties of plants^12^, which is more complex to understand. Because of the existence of pigments such as carotene and chlorophyll, reduced reflectance in the visible area is one feature of the vegetation’s spectral signature^13^. The red (600–700 nm) and blue (400–500 nm) bands in the spectrum are areas where chlorophyll absorption surges, whereas the NIR region has no absorbance^14^. Chlorophyll meters detect the leaf’s absorbance in the red and NIR region by utilizing these spectral characteristics. By utilizing these spectral characteristics in the both band, the meter quantitatively calculates chlorophyll levels in the leaf, helping to assess various stress conditions experienced by the plant^15^. Spectral signature of plant insisted upon (1) Growth stage (2) Environmental stresses and (3) Changes in irradiance^16^. The reliable way for deciding the plant’s N level is to use the reading from the chlorophyll meter, which precisely determines the relative chlorophyll content in maize leaves^17^. Since the performance of crop sensors is influenced by multiple factors beyond nitrogen such as weather, stress, and crop stage, a well-fertilized reference plot (or N-rich plot) is typically maintained to normalize sensor readings. To minimize variability in SPAD values and improve the reliability of nitrogen status assessment, we established a SPAD-based Sufficiency Index and an NDVI-based Response Index. These indices help neutralize the effects of confounding factors and enhance the precision of nitrogen recommendation decisions. Hence, the study strived upon the SPAD dynamics in maize and to determine its relationship with grain yield through correlation and regression analysis. Furthermore, multivariate analysis was performed alongside correlation and regression to identify the most reliable crop stage for nitrogen recommendation, thereby strengthening the decision-making process for precision nitrogen management.

## Materials and methods

### Location overview

**Fig. 1:**
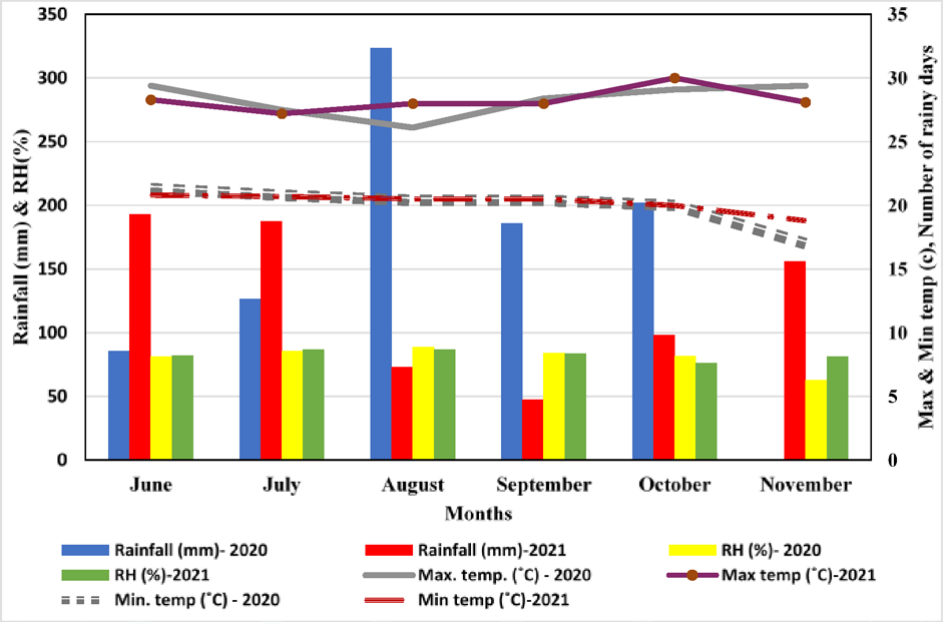
Monthly meteorological data during the crop growing period of the year 2020 and 2021 at the experiment site.

The field experiment was conducted at the Main Agricultural Research Station of the University of Agricultural Sciences in Dharwad, Karnataka, India, during the Kharif seasons of 2020 and 2021. Both years’ experiments were performed at the same geographical coordinates: latitude 15°30’6.0” N, longitude 74°59’13.2” E, and an altitude of 678 m above mean sea level. The research site recorded annual rainfall of 1012.9 mm in 2020 and 1052.6 mm in 2021, with July being the month of highest precipitation in both years. Adequate drainage measures were implemented to mitigate waterlogging effects. Rainfall was deemed sufficient and well-distributed for maize cultivation, supplemented by one additional irrigation in 2020 and two in 2021 under irrigated conditions. The variations in weekly and monthly minimum and maximum temperatures, along with relative humidity levels, did not exhibit significant impacts on crop growth and development. As shown in Fig. 1, in 2020 and 2021, the highest average monthly maximum temperature (36.3 °C and 36.7 °C, respectively) was in May and the lowest average monthly temperature (14.6 °C and 14.6 °C, respectively) was in December. The soil at the experimental site was characterized by a clay loam texture, with a pH of 7.65 (1:2.5). It exhibited a normal soluble salt content, indicated by an electrical conductivity (EC) of 0.31 dS m⁻¹. Nutrient analysis revealed low levels of available nitrogen at 258.4 kg ha⁻¹ and soil organic carbon at 0.45%. In contrast, the soil had high levels of available potassium at 367.65 kg K₂O ha⁻¹ and medium levels of available phosphorus at 34.35 kg P₂O₅ ha⁻¹. Phenological stages of maize at which the SPAD values were recorded have been mentioned in the Table 1.

**Table 1:** Phenological stages of maize at which the SPAD values were recorded.

Phenological stages	Description	Phenological stages	Description
V3	Collar of 3rd leaf visible	R1	Silking stage
V5	Collar of 5th leaf visible	R2	Blister stage
V7	Collar of 7th leaf visible	R3	Milk stage
V8	Collar of 8th leaf visible	R4	Dough stage
V10	Collar of 10th leaf visible	R5	Dent stage
V12	Collar of 12th leaf visible	R6	Physiological maturity
VT	Tasselling stage		

### Experimental frameworks and treatments

The experiment was set up using a strip plot design with three replications and total of sixteen treatment combinations each replicated three times. These arrangements comprised two distinct growing conditions (rain-fed and irrigated) organized in horizontal strips. They were combined with six precision nitrogen management practices, including nitrogen application based on SPAD sufficiency index (SI) ranges of 86–90%, 91–95%, and 96–100%, as well as Green Seeker-based response index (RI) ranges of 1.11–1.25, 1.26–1.40, and > 1.41, implemented in vertical strips (Table 2). These practices were applied alongside the recommended fertilizer doses (100:50:25 and 150:65:65 kg NPK ha^-1^ for rain-fed and irrigated conditions, respectively as recommended by the packages of practices of UAS, Dharwad), along with an absolute control, with each treatment replicated three times.

Maize hybrid NK-6240 was used for sowing. The amount of irrigation for M_2_ was 60 mm during 2020 and 120 mm during 2021. Each plot measured 24 m^2^ (6.0 m × 4.0 m). Entire K_2_O and P_2_O_5_ was applied as basal dose and nitrogen was guided to the crop from 15 DAS to 90 DAS as per SPAD and NDVI based indices. However, various factors may affect the SPAD chlorophyll meter and NDVI measurements^18^. Therefore, the SPAD based N sufficiency index (SI) and NDVI based response index (RI) approach has been used to isolate the effect of the N application on the SPAD and NDVI values of the crop from other factors that may affect the readings.

**Table 2:** Treatment imposed in the current research study plan.

Treatment details
A. Horizontal strips: growing conditions* • M_1_: Rain-fed • M_2_: Irrigated
B. Vertical strips: precision nitrogen management practices • T_1_: Nitrogen management through SPAD at sufficiency index of 86–90% • T_2_: Nitrogen management through SPAD at sufficiency index of 91–95% • T_3_: Nitrogen management through SPAD at sufficiency index of 96–100% • T_4_: Nitrogen management through GreenSeeker at response index of 1.11–1.25 • T_5_: Nitrogen management through GreenSeeker at response index of 1.26–1.40 • T_6_: Nitrogen management through GreenSeeker at response index of > 1.41 • T_7_: RDF (Recommended dose of fertilizer) • T_8_: Absoluteشcontrol

(a) Two nitrogen rich strip plots were maintained (application of 200% of RDN i.e., 200 and 300 kg N ha^–1^) for rain-fed and irrigated conditions, respectively.

(b) Under horizontal strip treatments (growing conditions*) the emphasis was on respective N recommendations to study spectral characterization and response to sufficiency and response index for N management.

(c) Irrigations were provided to irrigated condition strip based on rainfall during both the years.

(d) As reference readings, SPAD and GreenSeeker readings from both nitrogen-rich and absolute control plots were taken at 7-day intervals between 15 and 90 DAS, or from V3 to R4 stage.

### Crop management

Prior to crop sowing in all treatments except the absolute control plot, farm yard manure was added to the soil at a rate of 10 t ha^–1^ for irrigated conditions and 7.5 t ha^–1^ for rain-fed conditions. The recommended dose of fertilizer (RDF) was administered as urea, muriate of potash (MOP) and single super phosphate (SSP), respectively. For rain-fed agriculture, the RDF was 100:50:25 kg N PK ha^–1^. For irrigated agriculture, the RDF was 150:65:65 kg NPK ha^–1^. With the exception of absolute control, all treatments received the full application of P and K at the sowing time. For the rain-fed RDF treatment, 50% of the prescribed dose of N was delivered to the crop as a baseline application and the remaining 50% at 30 DAS (M_1_T_7_). 10% of the prescribed nitrogen dose was applied at the sowing time for the irrigated RDF treatment (M_2_T_7_), and the remaining nitrogen was applied in four splits at 20, 35, 50, and 65 DAS (20, 30, 30, and 10% RDN, respectively). In rest of the treatments (T_1_ to T_6_), urea was top dressed based on sufficiency index and response index approach (Tables 3 and 4). Across both years 2021 and 2022, each treatment received the same amount of nitrogen fertilizer as they did in the previous year. During *kharif* 2020 and 2021, SI of 96–100% under irrigated condition received a total of 251.3 kg (highest amount) of nitrogen in 8 splits at a rate of 33.75 kg ha^-1^ per application from 15 DAS up to 71 DAS, as and when the treatment attained respective sufficiency index (Tables 3 and 4). However, the time of application differed in the window of 15 DAS to 71 DAS during each year. The crop responded up to 251.3 kg of nitrogen, which is 67 per cent higher over the recommended dose of nitrogen (150 kg ha^-1^) under irrigated condition. To control the growth of the weeds, manual hoeing was used.

**Table 3:** The basal, split application and total amount of N fertilizer applied in different treatments (kg ha^− 1^) during 2020.

Treatments	Basal	15 DAS	22 DAS	29 DAS	36 DAS	43 DAS	50 DAS	57 DAS	64 DAS	71 DAS	78 DAS	85 DAS	Total (kg ha^− 1^)
Rainfed condition													
SI 86–90%	50	–	–	12.5	–	12.5	12.5	–	12.5		–	–	100.0
SI 91–95%	50	–	12.5	12.5	–	12.5	12.5	–	12.5	12.5	–	–	125.0
SI 96–100%	50	12.5	12.5	12.5	12.5	12.5	12.5	–	12.5	12.5	–	–	150.0
RI 1.1–1.25	50	–	12.5	–	12.5	12.5	12.5	–	–	12.5	–	–	112.5
RI 1.26–1.40	50	–	–	–	–	–	–	–	–	–	–	–	−50.0
RI > 1.41	50	–	–	–	–	–	–	–	–	–	–	–	−50.0
RDF	50	–	–	50	–	–	–	–	–	–	–	–	100.0
Control	0	–	–	–	–	–	–	–	–	–	–	–	0.0
Irrigated condition													
SI 86–90%	15	–	–	33.75	33.75	–	33.75	–	33.75	–	–	–	150.00
SI 91–95%	15	–	33.75	33.75	33.75	33.75	–	–	33.75	33.75	–	–	217.50
SI 96–100%	15	33.75	33.75	–	33.75	33.75	–	33.75	33.75	33.75	–	–	251.25
RI 1.1–1.25	15	33.75	33.75	–	33.75	33.75	–	–	33.75	–	–	–	183.75
RI 1.26–1.40	15	–	–	–	–	–	33.75	–	–	–	–	–	−48.75
RI > 1.41	15	–	–	–	–	–	–	–	–	–	–	–	−15.00
RDF	15	–	30	–	45	–	45	–	15	–	–	–	150.00
Control	0	**–**	**–**	**–**	**–**	**–**	**–**	**–**	**–**	–	–	–	0.0

**Table 4:** The basal, split application and total amount of N fertilizer applied in different treatments (kg ha^− 1^) during 2021.

Treatments	Basal	15 DAS	22 DAS	29 DAS	36 DAS	43 DAS	50 DAS	57 DAS	64 DAS	71 DAS	78 DAS	85 DAS	Total (kg ha^− 1^)
Rainfed condition													
SI 86–90%	50	–	–	12.5	–	12.5	–	–	12.5	12.5	–	–	100.0
SI 91–95%	50	–	12.5	–	12.5	–	12.5	12.5	12.5	12.5	–	–	125.0
SI 96–100%	50	12.5	12.5	–	12.5	12.5	12.5	12.5	12.5	12.5	–	–	150.0
RI 1.1–1.25	50	–	12.5	–	12.5	12.5	–	12.5	–	12.5	–	–	112.5
RI 1.26–1.40	50	–	–	–	–	–	–	–	–	–	–	–	50.0
RI > 1.41	50	–	–	–	–	–	–	–	–	–	–	–	50.0
RDF	50	–	–	50	–	–	–	–	–	–	–	–	100.0
Control	0	–	–	–	–	–	–	–	–	–	–	–	0.0
Irrigated condition													
SI 86–90%	15	–	–	33.75	33.75		33.75	–	33.75		–	–	150.00
SI 91–95%	15	–	33.75	33.75	–	33.75	33.75	–	33.75	33.75	–	–	217.50
SI 96–100%	15	33.75	–	33.75	–	33.75	33.75	33.75	33.75	33.75	–	–	251.25
RI 1.1–1.25	15		33.75	–	33.75	33.75	–	33.75	33.75	–	–	–	183.75
RI 1.26–1.40	15	–	–	–	–	–	–	33.75	–	–	–	–	48.75
RI > 1.41	15	–	–	–	–	–	–	–	–	–	–	–	15.00
RDF	15	–	30	–	45	–	45	–	15	–	–	–	150.00
Control	0	**–**	**–**	**–**	**–**	**–**	**–**	**–**	**–**	–	–	–	0.0

### Computations of related metrics

#### Sufficiency index (SI) value approach

Five randomly selected plants in each plot were used to provide the third completely developed leaf for the purpose of taking SPAD meter values. These readings were obtained from nitrogen-rich plots receiving 200% of the recommended dose of nitrogen (200 and 300 kg N ha^-1^ for rain-fed and irrigated conditions, respectively). Subsequently, these readings were utilized to compute the SPAD sufficiency index (SI). The sufficiency index is determined by calculating the ratio of the SPAD value recorded from the test plot, using a SPAD 502 m, to the SPAD value of an over-fertilized reference plot (N Rich plot), expressed as a percentage^19^.

1 $$\:N\:Sufficiency\:Index\:\left(SI\right)=\frac{SPAD\:value\:of\:test\:plot}{SPAD\:value\:of\:N\:rich\:plot}$$

#### Response index (RI) value approach

RI is derived from NDVI values (recorded using GreenSeeker) which is an inverse of SI. The Response Index is computed as the ratio between the NDVI value observed in the nitrogen-rich reference plot (often referred to as the “N rich plot”) and the NDVI value recorded in the test plot^19^.

2 $$\:N\:Response\:Index\:\left(RI\right)=\frac{NDVI\:value\:of\:N\:rich\:plot}{NDVI\:value\:of\:test\:plot}$$

Fertilizer N was top dressed at 25 per cent of RDN ha^-1^ when RI or SI value fall in the set range (T_1_ to T_6_ treatments).

### Collection of SPAD meter readings

The third fully expanded leaf from the top of the plant was used to record SPAD meter readings. The observations were recorded from 15 DAS to 90 DAS i.e., V3 to R4 stage with an interval of 7 days. A total eleven observations were recorded throughout the crop growth period at different phenological stages (V3 to R4 stage) during both the years. The SPAD readings were obtained from five plants in each plot, with three readings from the midsection of each leaf. All the SPAD meter readings were averaged and expressed as a SPAD value of a plot.

### Yield determination

At maturity stage, cobs from each net plot were harvested. Cobs were dehulled, air dried, shelled, cleaned and weighed. Per plot yield was quantified and it is converted to yield per ha and expressed in kg ha^-1^.

### Unveiling of data insights using statistical tool

The experimental data obtained at different growth stages were compiled and subjected to statistical analysis by adopting Fischer’s method of analysis of variance technique for strip plot as outlined by Gomez and Gomez^20^. At 5 per cent level of significance ‘F’ test was carried out and the critical difference (CD) values were calculated wherever ‘F’ test was significant. Correlation matrix was constructed between the SPAD data and grain yield using the package “corrplot” in R-Software version 4.4.0. Simple linear regression was attended to calculate the coefficient of determination for yield with SPAD values using Microsoft Excel^20^. The PCA analysis done using the statistical package KAU grapes website^21^.

## Results: SPAD dynamics of maize

### ANOVA of SPAD chlorophyll meter readings collected at different growth stages

SPAD readings were collected at 15 (V3), 22 (V5), 29 (V7), 36 (V8), 43 (V10), 50 (V12), 57 (VT), 64 (R1), 71 (R2), 78 (R3) and 85 DAS (R4 stage). In totality, 11 SPAD readings were taken throughout the crop growth period. No significant differences were observed across the replications. With the exception of 15, 43, 57, 71, 78, and 85 DAS (*p* < 0.05), growing conditions (Factor A) has no significant effect on the maize SPAD dynamics at any stage of crop growth (Table 5). SPAD sufficiency index-based treatments provided need-based nitrogen application (Factor B) and recorded significantly higher SPAD values as compared to the blanket recommendations. For the interaction effect (growing conditions vs. precision nitrogen management practices), significant differences in SPAD readings were observed at all stages of crop growth, except for V5, VT, R1, and R4 stages (*P* = 0.05). Study revealed that SPAD readings increased with increasing nitrogen application.

**Table 5:** Analysis of variance (ANOVA) for SPAD readings (15 to 85 days after sowing at 7-day intervals) affected by precision nitrogen management practices and growing conditions.

	Mean sum of squares											Yield (kg ha^− 1^)
ANOVA	15شDAS	22شDAS	29شDAS	36شDAS	43شDAS	50شDAS	57شDAS	64شDAS	71شDAS	78شDAS	85شDAS	
Stage of the crop	V3 stage	V5 stage	V7 stage	V8 stage	V10 stage	V12شstage	VTشstage	R1شstage	R2شstage	R3شstage	R4شstage	
Replication	4.17	0.39	3.68	1.88	1.60	3.87	3.11	0.27	0.01	15.38	2.47	21484.9
Factor A (growing conditions)	31.46*	26.17 ^NS^	1.01 ^NS^	15.12 ^NS^	32.15*	6.38 ^NS^	17.19*	14.25 ^NS^	70.73*	153.81*	61.95*	14131918.4*
Error (A)	1.41	2.29	0.91	1.96	0.67	0.47	0.89	2.90	1.36	7.50	0.52	1477.4
Factor B (precision nitrogen management practices)	54.25*	68.74*	143.14*	129.92*	179.63*	188.34*	444.20*	432.04*	527.07*	417.54*	404.59*	31642917.1*
Error (B)	1.25	1.71	1.35	0.83	1.26	0.55	1.09	1.62	1.63	3.84	2.08	105944.5
Interaction (factor A*B)	5.10*	4.20	7.93*	2.43*	8.62*	8.35*	4.39	2.65	3.21*	9.46*	4.33	2905132.0*
Interaction (c)	1.04	1.96	1.93	0.42	0.61	1.19	2.01	2.48	0.58	0.92	1.70	87914.1

*Significant at 0.01 level of probability.

^NS^non significant.

### Correlation analysis of SPAD values with grain yield of maize under rainfed and irrigated condition

**Fig. 2:**
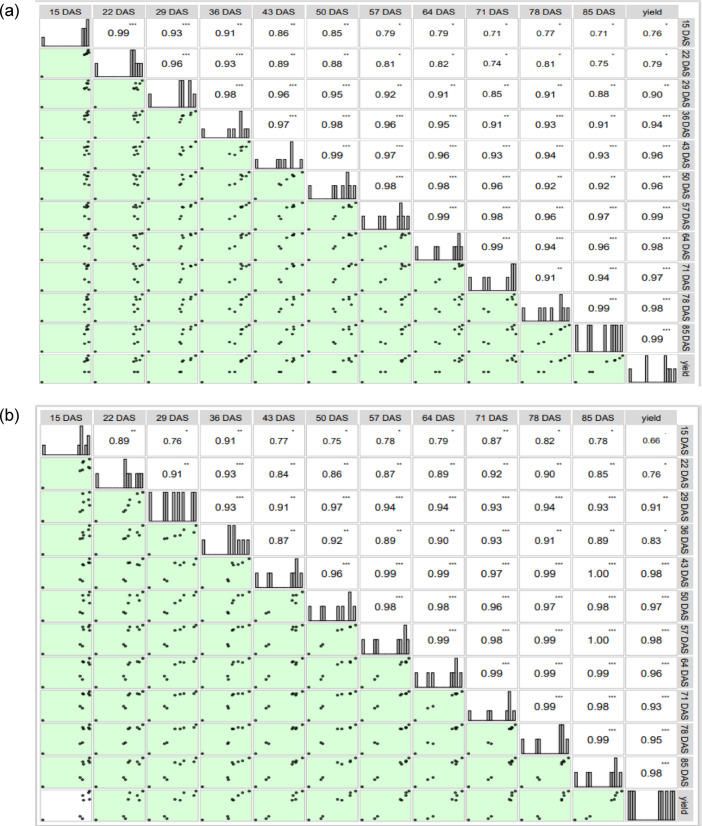
(**a**,**b**) Correlation matrix of SPAD at various growth stages and grain yield readings in Maize under rainfed (**a**) and irrigated condition (**b**). *, ** and *** indicate significance at 10, 5 and 1%, respectively, (Confer Table 5 for crop growth stages).

Figure 2a, b shows the Karls Pearson’s correlation coefficient between the SPAD values recorded at various growth phases and yield under rainfed and irrigated conditions, respectively. A robust and positive connection was observed between the SPAD values and yield under rainfed condition. The SPAD readings were collected at the growth stages after V5 stage, positively correlated with grain yield. Week and positive correlation between yield and SPAD values was observed at V3 (15 DAS) and V5 stage (22 DAS) as compared SPAD readings collected at subsequent growth stages (*p* < 0.01, 0.05). Whereas, significantly higher correlation between SPAD values and yield was noticed at VT (*r* = 0.99), R1 (*r* = 0.98), R3 (*r* = 0.98) and R4 stage (*r* = 0.99) at one per cent level of significance.

Positive correlation was found between SPAD values and yield under irrigated condition similar to rainfed condition. Degree of correlation (r) between SPAD readings and seed yield revealed an increasing trend from 43 to 85 DAS indicating strong positive correlation with the yield and SPAD values, under irrigated condition. High correlation coefficients with a high level of significance (*p* < 0.01) were detected at 43 DAS (*r* = 0.98), 57 (*r* = 0.98) and 85 DAS (*r* = 0.98). Week correlation were observed at V3 and V5 stages of the crop growth under irrigated condition.

### Regression analysis of SPAD values with grain yield of maize

**Fig. 3:**
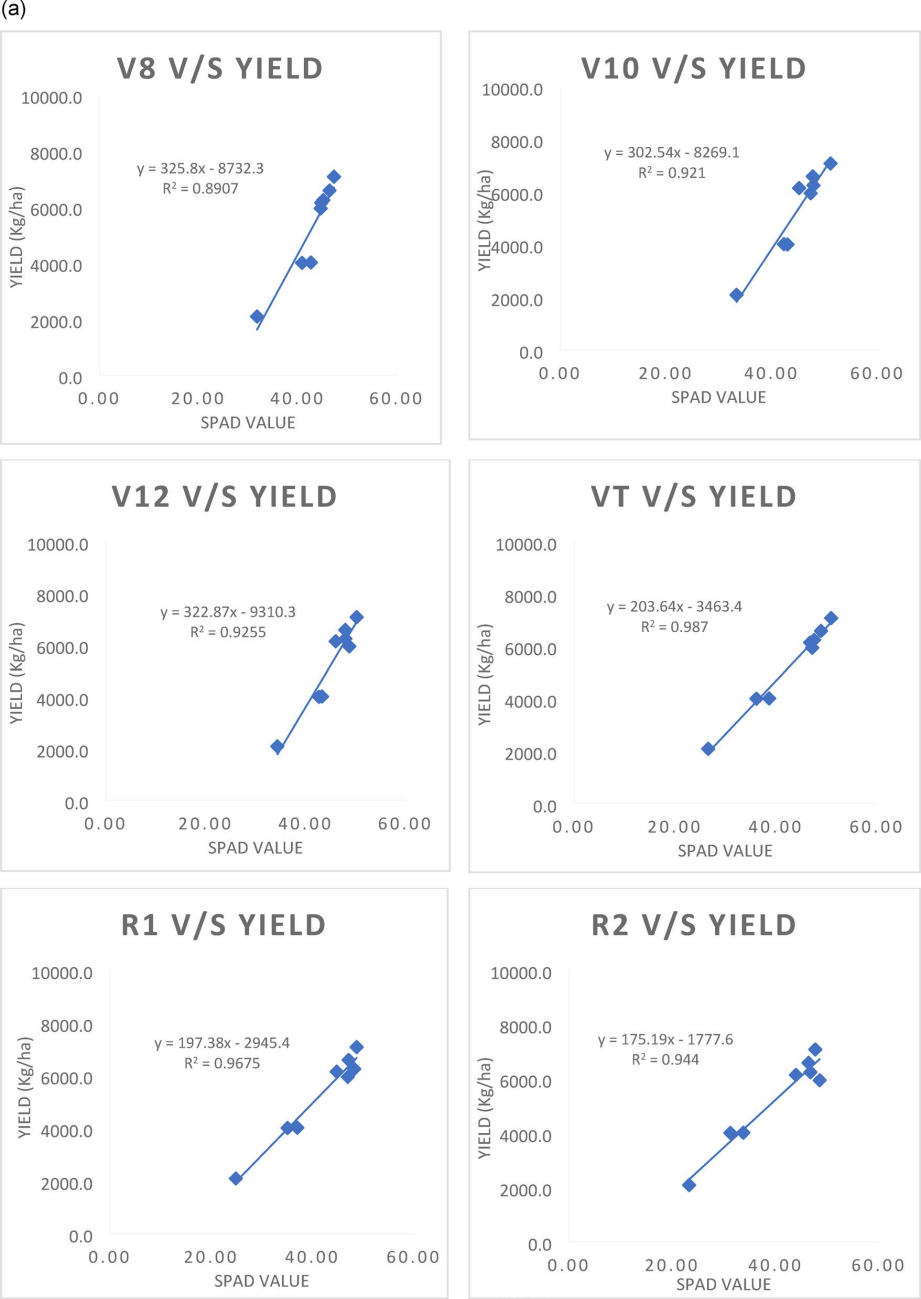

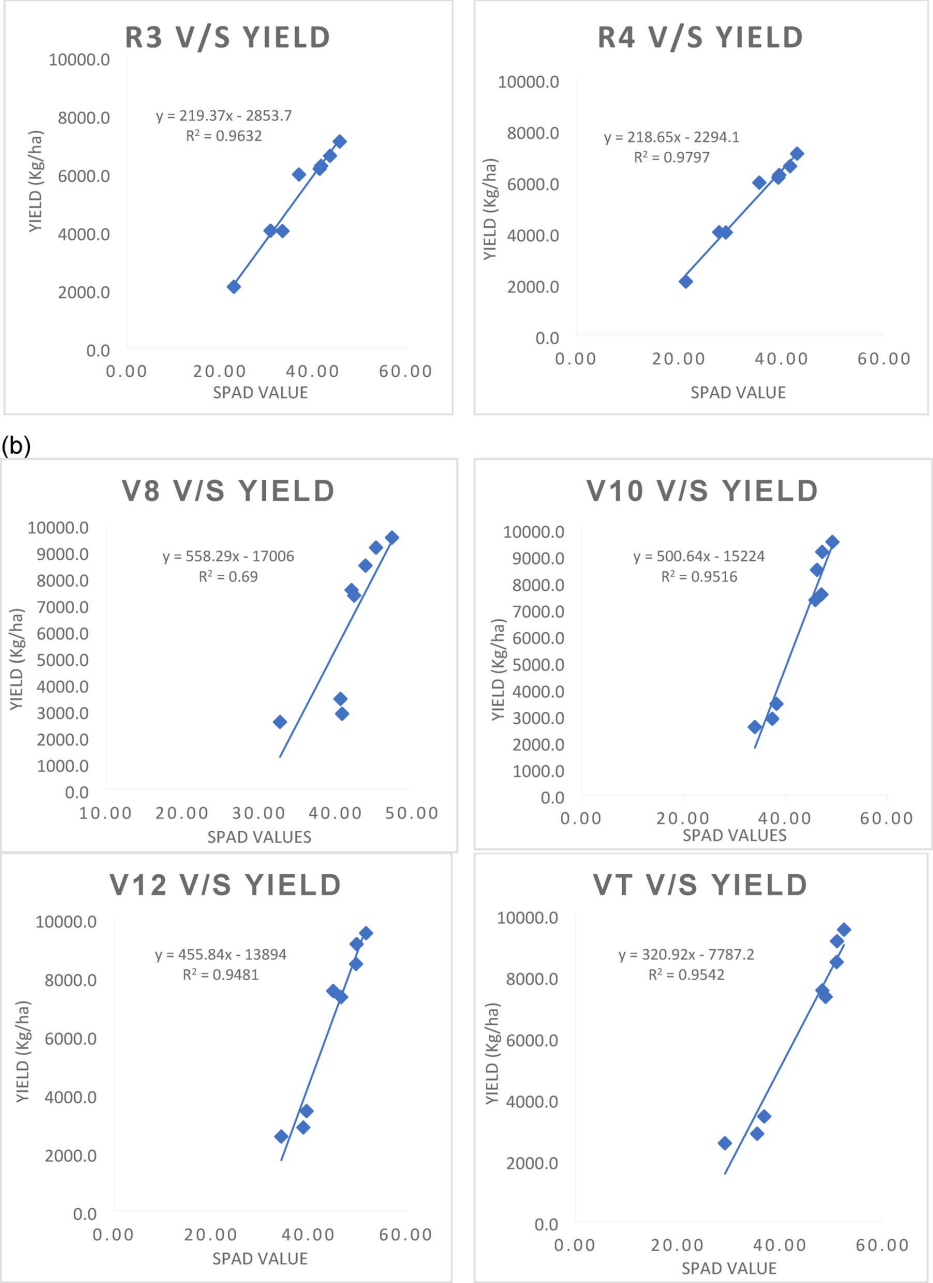

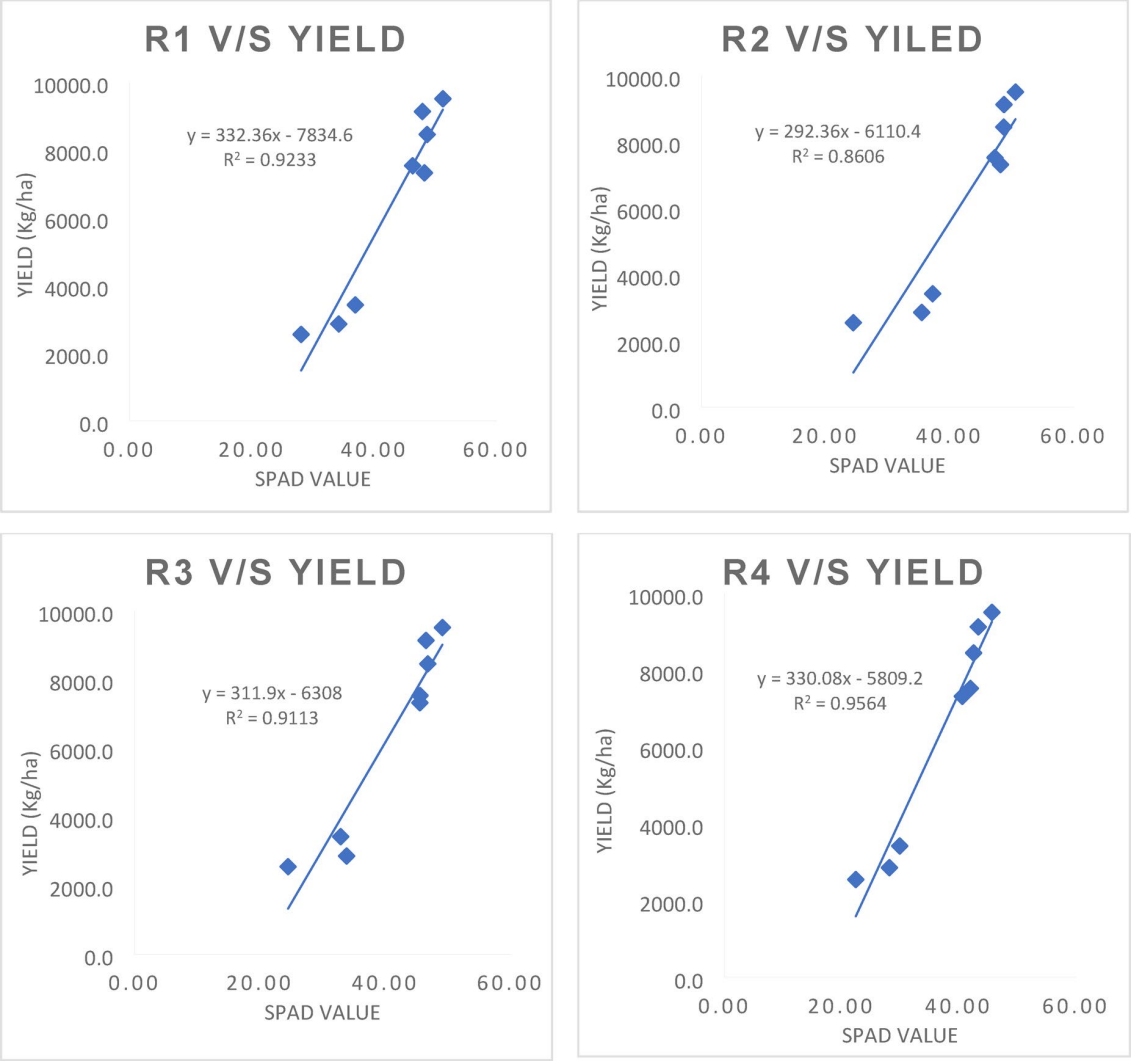
(**a**) Regression analysis of SPAD values at various growth stages with grain yield of maize under rainfed condition. (**b**) Regression analysis of SPAD values with grain yield at various growth stages of Maize under irrigated condition.

The simple linear regression was worked out between independent variable i.e., SPAD values recorded at various growth stages (V8 stage to R4 stage) with grain yield (dependent variable as Y) as affected by precision nitrogen management practices and growing conditions are presented in Fig. 3a, b. Under the rainfed condition, significant positive linear correlation was observed between SPAD values and maize grain yield. Linear regression equations worked out at different growth stages of crop under rainfed condition are given below.

**Table:** 

Stage	Mathematical model	R^2^
V8 stage	y = 325.8x−8732.3	0.89
V10 stage	y = 302.54x−8269.1	0.92
V12 stage	y = 322.87x−9310.3	0.93
VT stage	y = 203.64x−3463.4	0.99
R1 stage	y = 197.38x−2945.4	0.97
R2 stage	y = 175.19x−1777.6	0.94
R3 stage	y = 219.37x−2853.7	0.96
R4 stage	y = 218.65x−2294.1	0.98

Under rainfed condition, the optimal model has performed better and chosen based on the R^2^ value, nearly 99 and 98 per cent of variation in grain yield is explained by this variable (SPAD) at VT and R4 stage, which were followed by R1 and R3 stage with the R^2^ value of 0.97 and 0.96, respectively (*p* < 0.05).

Similar to the above case, under the irrigated condition, various linear regression equations were formulated by including SPAD readings from 36 (V8 stage) to 85 DAS (R4 stage). Linear regression equations worked out at different growth stages of crop under irrigated condition are as follows:

**Table:** 

Stage	Mathematical model	R^2^
V8 stage	y = 558.29x−17006	0.69
V10 stage	y = 500.64x−15224	0.95
V12 stage	y = 455.84x−13894	0.95
VT stage	y = 320.92x−7787.2	0.95
R1 stage	y = 332.36x−7834.6	0.92
R2 stage	y = 292.36x−6110.4	0.86
R3 stage	y = 311.9x−6308.1	0.91
R4 stage	y = 330.08x−5809.2	0.96

The equations containing SPAD value at V10, V12, VT and R4 stage were shown higher R2 value of 0.95, 0.95, 0.95 and 0.96, respectively and arbitrated as the best model under irrigated condition. From this linear regression model, a high significance (*p* < 0.05) for SPAD chlorophyll readings at 43, 50, 57 and 85 DAS was observed, which corresponded to the early bloom stage and early maturity stage of the crop.

### Relation between observed and predicted yields

**Fig. 4:**
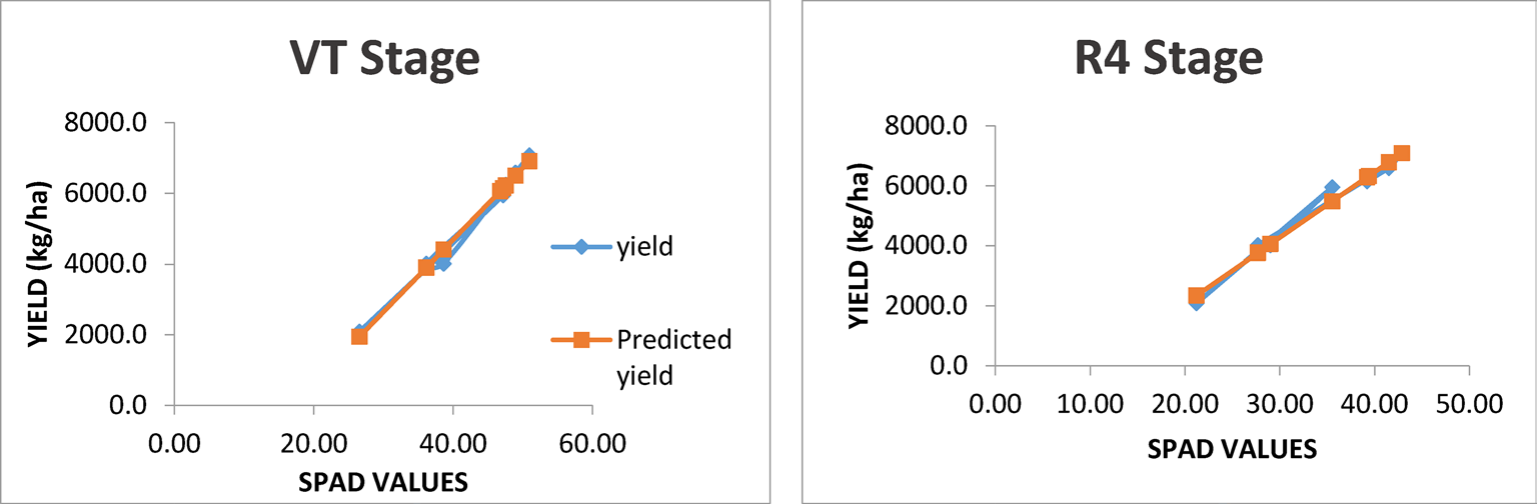
Relation between observed yield and predicted yield developed using SPAD values recorded at VT and R4 stage under rainfed condition.

**Fig. 5:**
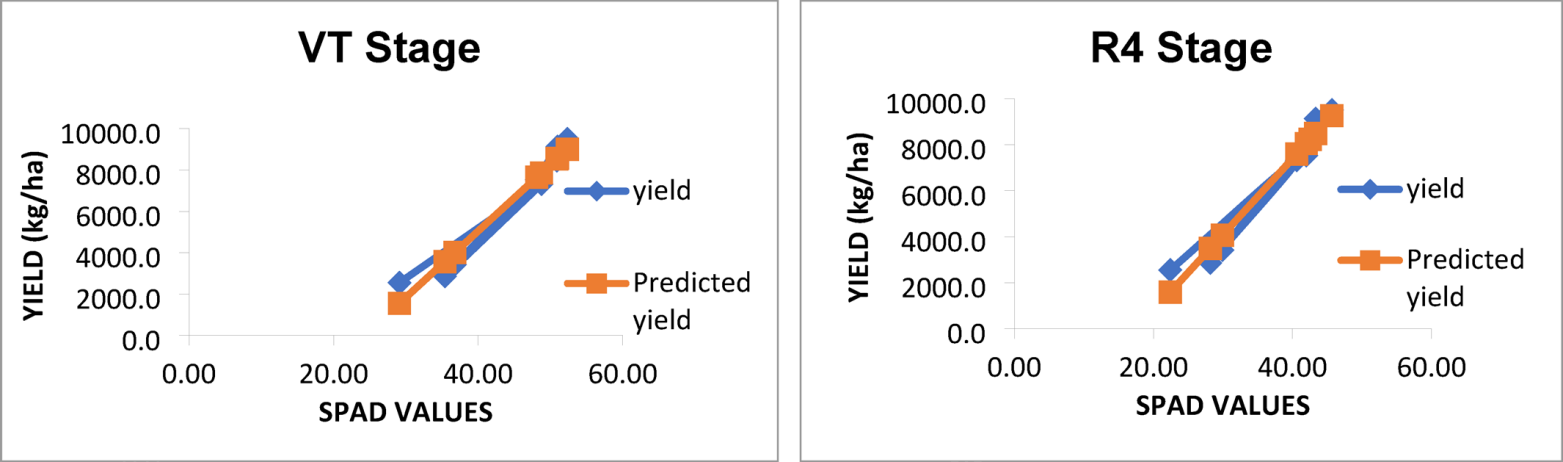
Relation between observed yield and predicted yield developed using SPAD values recorded at VT and R4 stage under irrigated condition.

The graphs showing observed and predicted yield using the models with best R^2^ value are presented in Figs. 4 and 5. Under both rainfed and irrigated conditions, models predicted accurately with the least difference between observed and predicted yield (Figs. 4 and 5, respectively) developed using SPAD values at VT and R4 stages.

### The impact of varying growing conditions and the implementation of precision nitrogen management techniques on the grain yield of maize

**Fig. 6:**
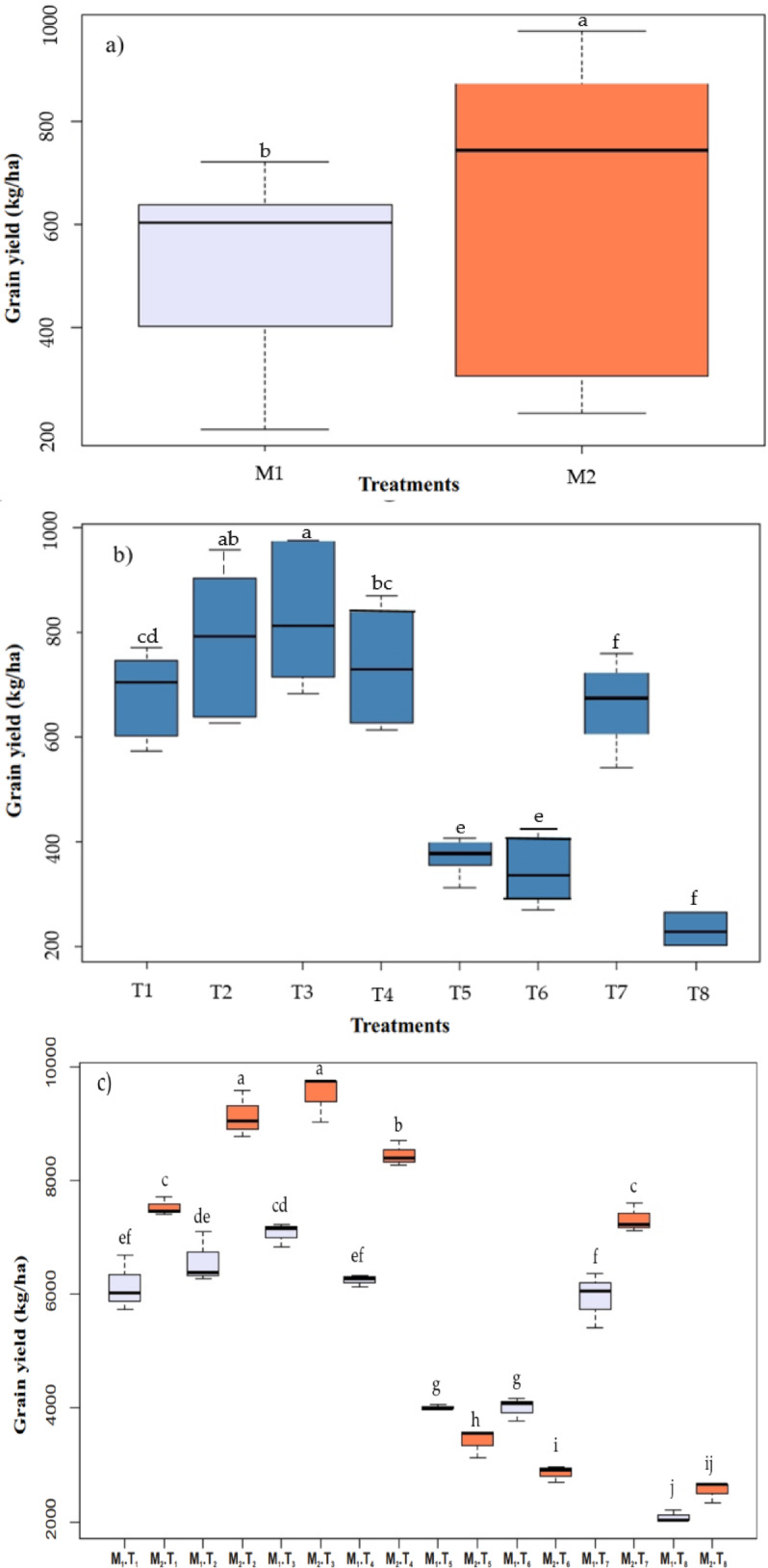
(**a**,**b**) Influence of growing conditions (**a**) and precision nitrogen management practices (**b**) on grain yield of maize. (**c**) Impact of interaction between precision nitrogen management practices and growing conditions on grain yield of maize.

Figure 6 illustrates the substantial influence of precision nitrogen management practices on the grain yield under rain-fed and irrigated conditions. Maize grain yield presented in Fig. 6a indicates that maize grown under irrigated condition produced significantly higher grain yield (6346.7 kg ha^-1^) than rain-fed condition (5261.5 kg ha^-1^). With the exception of nitrogen application at SI of 91–95% (7858.0 kg ha^-1^) when compared with the absolute control (2319.2 kg ha^-1^), significantly higher grain yield (8291.9 kg ha^-1^) was recorded in the treatment consisting of nitrogen management through SI of 96–100% as compared to other treatments (Fig. 6b).

Grain output was significantly impacted by both growth conditions and specific nitrogen management practices (Fig. 6c). Notably, compared to the other treatments, growing maize under irrigation and applying nitrogen at a sufficiency index (SI) of 96–100% (M_2_T_3_) produced significantly higher grain yield (9508.2 kg ha^–1^). However, it was statistically on par to the nitrogen application at SI of 91–95% under irrigation condition (9131.3 kg ha^–1^).

### Multivariate analysis of the SPAD dynamics

**Fig. 7:**
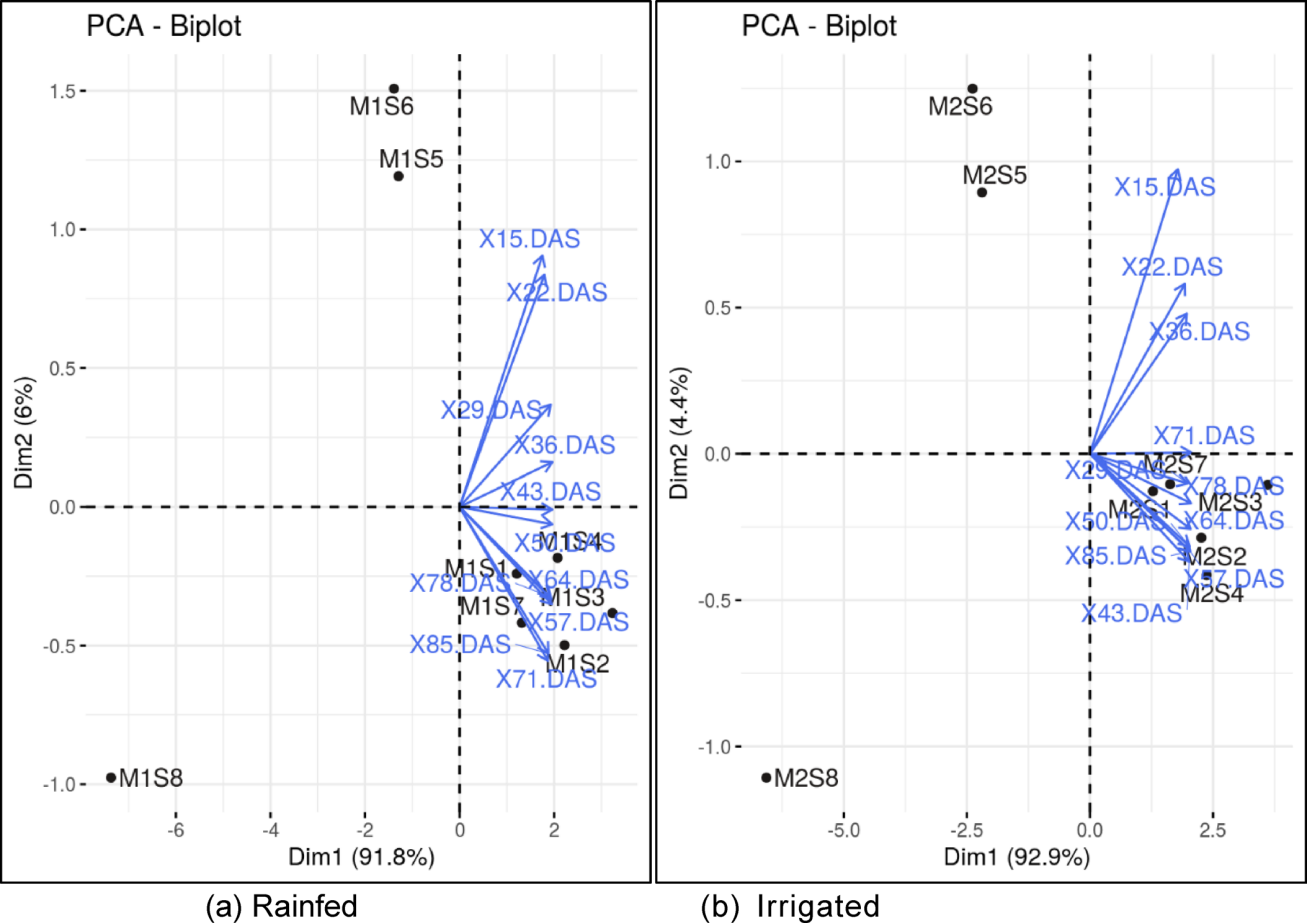
Biplots of PCA of rainfed (**a**) and irrigated condition (**b**) under various precision nitrogen management practices.

Soil moisture content plays a crucial role in influencing PCA loading in maize by affecting various physiological and growth parameters. The only components which have the variance more than 5 are chosen as the principle components (While choosing the PC’s either one can choose eigen value > 1 or percentage of variance > 5). Among different water regimes, PC1 comprised of knee high stage, and contributed to 91.82% to the variation, and PC2 comprised of only V3 stage by which PC2 contributed 6.032% to variation and total percent of variation contributed by both the PC’s is 97.86% to the total variation in rainfed condition. However, in irrigated condition, there is 1 principle component which is contributing much to the variation (71 DAS), The correlation coefficient between the chosen variables is > 0.6 (Fig. 2; Fig. 7) and avoided those stages which are having higher correlation of more than 0.6.

**Table 6:** Summarization of PCA under rainfed and irrigated conditions.

Principle component	PC1	PC2	PC1
Eigenvalue	10.10	0.66	10.21
Percentage of variance	91.82	6.03	92.87
Cumulative percentage of variance	91.82	97.86	92.87
Factor loadings			
SPAD reading stage (DAS)	Rainfed		Irrigated
15	–0.278	**–0.56**	–0.27
22	–0.285	–0.518	–0.291
29	–0.306	–0.228	–0.300
36	**–0.311**	–0.1	–0.297
43	–0.310	0.006	–0.305
50	–0.310	0.039	–0.306
57	–0.309	0.217	–0.308
64	–0.308	0.206	–0.309
71	–0.297	0.344	**–0.311**
78	–0.302	0.2	–0.310
85	–0.298	0.327	–0.307

Bold faced values are the principle components and underline values are the principle components chosen based on the correlation.

## Discussion

### Correlation between SPAD values and growth parameters, yield parameters, yield and nutrient uptake of maize

The correlation graph revealed that SPAD values reflecting crop N status is strongly and positively correlated with yield under both growing conditions. The SPAD Values increased with growth stages until VT or R1 stage, later decreased gradually.

The correlation between SPAD readings and maize yield (Fig. 2a) is typically stronger under rainfed condition^22,23^. Under rainfed conditions, maize plants often experience water stress, which can lead to physiological changes that affect leaf greenness. The SPAD index, which measures chlorophyll content, serves as a reliable indicator of plant health and stress levels. Study has shown that higher SPAD values correlate well with increased yield under these environments because they reflect plant’s ability to cope with stress and maintain photosynthetic efficiency^23–26^. In irrigated environments, where water is consistently available, the relationship between SPAD values and maize yield (Fig. 2b) may be less pronounced, leading to a weaker correlation between SPAD readings and yield. Studies have shown that while SPAD values still correlate with yield in irrigated conditions, the strength of this correlation is often reduced compared to rainfed scenarios^27,28^. The presence of optimal irrigation levels can enhance growth and yield but may not always translate to a strong relationship with SPAD readings due to more stable growing conditions^29^.

Less variance in spectral features among nitrogen applied treatments may have resulted from lower N demand and adequate N supply to meet crop requirements, as seen by the lower correlation values during the V3 and V5 growth stages compared to other growth stages. As a result, using spectral data from these stages of growth to forecast yield would be unrealistic. The results are consistent with Kandel^30^ found that SPAD values rise with increase in corn growth stages. At the R1 and R2 stages, a positive correlation between grain yield and SPAD values was noted. Later in the reproductive growth stages, notably at R4 and R5, Piekielek et al.^31^ and Blackmer et al.^32^ observed a greater association between SPAD meter readings and maize grain yield. Solari et al.^33^ reported a stronger correlation between chlorophyll meter readings at the R1, R2, and R3 stages with yields. When Simko, A. and Veres^34^ measured the SPAD values in the R1 stage, they found a good association (*r* = 0.966) between the SPAD levels and yield. According to Rostami et al.^35^, there is a positive and significant rise in the link between SPAD and maize grain yield during the reproductive stage of crop growth.

In a similar vein, Rorie et al.^36^ also showed that there was a greater correlation between yield and SPAD measurements recorded during the VT stage, with r values of 0.90. Milagres et al.^37^ noticed that significant correlation exists between the SPAD readings and leaf N content at V10, V12 and R1 stages.

### Regression analysis of SPAD values with grain yield of maize

The high R² values observed at the VT and R4 stages under rainfed condition suggest that SPAD readings are particularly effective at predicting maize yield when plants are under stress due to limited water availability. Evidently, least difference was observed between observed and predicted yield as depicted in Figs. 4 and 5. This is likely because chlorophyll content, as indicated by SPAD readings, directly reflects the plant’s ability to photosynthesize and cope with stress. Under such conditions, any variation in chlorophyll content can significantly impact yield outcomes. Szeles et al.^38^ found that the combined SPAD and Leaf Area Index (LAI) provided a strong prediction model for maize yield, with the highest correlation observed at the VT stage (R² = 0.762).

In irrigated environments, while the R² values remain high (0.95 to 0.96), they are slightly lower than those observed under rainfed condition. This difference may be attributed to the more stable growing environment provided by irrigation, which reduces stress on the plants. As a result, variations in SPAD readings may not translate as directly into yield differences. SPAD values correlate well with maize yield in both irrigated and rainfed environments, the strength of this correlation is generally higher in rainfed systems due to increased plant stress. The timing of SPAD measurements is crucial in both water regimes. The VT stage is particularly critical as it marks a transition to reproductive growth when nutrient demands increase dramatically. Similarly, the R4 stage coincides with kernel development, making chlorophyll content at these stages a vital indicator of potential yield. Research has shown that chlorophyll content is closely linked to grain filling and overall biomass accumulation during these key growth phases^25,38^.

The outcomes are consistent with Blackmer and Schepers’ (1995) research, which showed that yield and SPAD reading at the R4 (dough stage) had a higher correlation and that SPAD measurements at the V6 stage had a significant correlation to final grain yield (*p* < 0.01) but poor predictability (R^2^ = 0.25). Similarly, Lindsey et al.^39^. and Rostami et al.^35^. revealed from the regression analysis that SPAD values at R1 stage accurately predicted grain yield, indicating that photosynthetic maturity represents the optimal time for predicting nitrogen status in corn. Although the yield forecast based on readings at V6 demonstrated relevance for SPAD value, the deviations explained were not well suited to be used in recommending nitrogen application rates in the future. According to Singh and Singh^40^, SPAD values are a reliable indicator of spring maize’s in-season nitrogen need as well as grain yield. Grain yield in the V9 stage was predicted by SPAD values, which also explained 62% of the variability, which increased to 70% at the V12 stage and 75% at the VT stage.

### The impact of varying growing conditions and the implementation of precision nitrogen management techniques on the grain yield of maize

Higher yield under irrigated condition might be due to the optimum moisture throughout the growing period and split application of nitrogen which led to adequate availability of nitrogen during the crop growth period resulted in higher leaf water potential and photosynthesis, owing to increased dry matter production. Bello^41^ noticed that grain yield was higher under rainfed condition with supplemental irrigation as compared to rainfed condition alone, which increased the accumulation of photosynthates and finally increased the yield. The findings of Majid et al.^42^, Kresovic et al.^43^ and Kumar et al.^44^ are similar to that of obtained results.

Grain yield of the maize was significantly influenced by precision nitrogen management practices (Fig. 6b). The higher grain yield of maize was mainly attributed to the precise application of nitrogen during the vegetative stage of plant growth led to the better translocation of carbohydrate reserve from source to sink resulting in higher total dry matter production. The need-based side dressing of nitrogen after anthesis stage resulted in higher post anthesis nutrient uptake that led to the significant increase in yield attributes and finally the grain yield. Research revealed that SPAD based precision nitrogen management in maize has produced higher grain yield over control and RDF^45^. These results are in conformity with the findings of Rostami et al.^35^ and Kumar et al.^46^ in maize.

Among interactions, growing maize under irrigated condition with the application of nitrogen at SI of 96–100% (9508.2 kg ha^− 1^) recorded significantly higher grain and straw yield as compared to the rest of the treatments (Fig. 6c). Higher application of nitrogen under SPAD based nitrogen application treatments, which synchronized with the crop demand at V3, V5 and V8 stage led to the greater uptake, improved photosynthesis and proper partitioning of the photosynthates to sink resulted in higher dry matter production. Post-anthesis application of nitrogen (at V12/VT and R1 stage) have led to the maintenance of higher green leaf area per plant and translocation of assimilates towards reproductive parts resulted in higher yield attributes^47^. Maximum yields in irrigated maize were attained when early season N levels were maintained adequate between sufficiency indices 90 and 100 per cent at the V8 growth stage^48^.

### Multivariate analysis of SPAD dynamics under different water regime

High factor loading of the PC indicated that V3 (15 DAS) was the stage when applied fertilizers based on the sufficiency index and response index and might have been efficiently used by the crop to put forth the productivity in rainfed condition, however in irrigated condition, application of fertilizer at V12 stage could be most effective. Higher moisture levels improve nutrient availability and uptake, leading to enhanced growth traits, which are often reflected in PCA as high loadings on principal components^49^. Studies have shown that moisture content has a strong correlation with grain yield and other important traits like chlorophyll content, especially under different moisture regimes^50^. Therefore, PCA effectively captures the relationship between soil moisture and maize performance by highlighting the essential traits.

### Limitations of the study

A limitation of this study is the potential variability in SPAD readings caused by environmental factors such as light intensity, temperature, and plant factor (leaf position), which may affect the accuracy of chlorophyll measurements. While strong correlations between SPAD values and yield are noted at certain growth stages, the variability in SPAD readings at earlier stages limits their predictive accuracy. The study’s reliance on SPAD readings alone may not fully capture the complexities of nitrogen dynamics in the soil-plant system.

### Areas for further research

Future research could integrate SPAD readings with other non-destructive sensors and remote sensing technologies to enhance the precision of nitrogen status assessment and yield prediction in maize. Additionally, exploring the impact of varying environmental conditions and soil types on SPAD readings and their correlation with maize growth parameters could provide more robust and adaptable nitrogen management strategies.

## Conclusion

Chlorophyll content is one of the major parameters that determine photosynthetic activity and has a significant impact on yield. Chlorophyll plays a crucial role in photosynthesis, directly influencing crop development, yield and photosynthetic efficiency. The results showed that, maize cultivated under irrigation and nitrogen applied at a SPAD-based sufficiency index (SI) of 96–100% produced a significantly greater grain yield, with the exception of nitrogen applied at a SI of 91–95% under irrigated condition. A robust and positive relationship was observed between the yield and SPAD values documented at different stages of maize growth. SPAD readings collected after V5 stage were positively correlated with grain yield and higher correlation was noticed at VT, R3 and R4 stage and week correlation was observed at V3 and V5 stage (*p* < 0.01, 0.05).

There is a significant positive linear relationship between SPAD and maize grain yield, according to the results of the basic linear regression analysis. Models developed using SPAD values predicted yield accurately with the least difference between observed and predicted yield. Through maintaining higher chlorophyll content during late vegetative stage and early reproductive phase of crop growth through nitrogen application would be beneficial in increasing the grain yield of maize irrespective of the growing conditions. Multivariate analysis clearly indicated that the important management stages (V5 and V3 in rainfed and V12 in irrigated condition) to fetch higher yield in maize, which doesn’t mean that subsequent growing stages aren’t important, but the highlighted stages need to managed precisely.

Therefore, it can be inferred by use of predictive analysis of yield through correlation and regression tools as well as multivariate analysis, combined with precision nitrogen management in different growing conditions is a better way for farmers. It is a cost effective and resource saving technology. While, two season study can provide valuable insights and serve as a strong foundation for understanding the key trends. However, to derive broader recomendations, comprehensive research conducted over multiple seasons and diverse climatic conditions by integrating remote sensing technologies would help strengthen and enhance the reliability and applicability of the findings.

## Data Availability

The datasets used and/or analyzed during the current study are available from the corresponding author upon reasonable request.
